# ESKAPE pathogens: Emerging isolates in odontogenic orofacial infections

**DOI:** 10.1016/j.jobcr.2026.101497

**Published:** 2026-07-31

**Authors:** P.S. Tripthi, Rakshita Kumar, Padmaraj Hegde

**Affiliations:** Nitte (Deemed to Be University), AB Shetty Memorial Institute of Dental Sciences (ABSMIDS), Department of Oral and Maxillofacial Surgery, Mangalore, India

**Keywords:** Oro-facial infection, ESKAPE pathogens, Drug resistance

## Abstract

**Introduction:**

Odontogenic orofacial infections involve a broad microbial spectrum, with the rise of multidrug-resistant organisms complicating management. ESKAPE pathogens, known for their ability to evade standard antimicrobial therapies, are increasingly implicated in severe infections. However, their prevalence and resistance patterns in odontogenic orofacial space infections remain insufficiently reported. This study aimed to identify the occurrence of ESKAPE pathogens in such infections and assess their antimicrobial susceptibility to guide effective treatment.

**Methodology:**

Between August 2019 and June 2022, 132 patients with oral and maxillofacial infections were prospectively enrolled. Pus samples collected using sterile swabs were cultured following standard protocols. Bacterial identification and susceptibility testing were performed using the VITEK 2-Compact system.

**Results:**

Twenty-nine samples (21.97%) yielded at least one ESKAPE pathogen, with Enterobacter species being the most common isolate (34.48%). High resistance rates were seen to amoxicillin–clavulanic acid (62.07%), cefuroxime (62.5%) and trimethoprim–sulfamethoxazole (68.42%). Linezolid, ertapenem, and clindamycin demonstrated the highest susceptibility (100%), followed by imipenem (86.96%, p ≤ 0.05). *Acinetobacter baumannii* demonstrated complete resistance to all tested antibiotics.

**Conclusion:**

ESKAPE pathogens represent a significant proportion of odontogenic orofacial infections and exhibit considerable resistance to commonly used antibiotics. These findings reinforce the necessity of culture-based therapy and highlight imipenem as a potential option for resistant cases. Strengthened antibiotic stewardship and continued surveillance are essential to manage the growing threat of multidrug-resistant organisms in maxillofacial infections.

## 1 Introduction

Odontogenic oro-facial space infection has been prevalent and reported since as early as the second century.^1^ With the advent of antibiotics, Penicillin paved the way for treatment of such infections. However the ever evolving bacteriological spectrum of the oral flora towards more resistant strains has enhanced the potential for grave outcomes of dental infections. These infections are frequently multifactorial, caused by amalgam of aerobic and anaerobic bacteria and are further complicated by superadded nosocomial infections^2^^,^^3^ the past decade has seen a major shift in trends in the prevalence of non-native and unusual causative micro biomes.^3^ Particularly problematic are the ESKAPE pathogens: notorious multidrug-resistant bacteria, infamous for their ability to ‘escape’ the effects of antibiotics 4, 5, 6. ESKAPE pathogens include *Enterococcus faecium*, *Staphylococcus aureus*, *Klebsiella pneumoniae*, *Acinetobacter baumannii*, *Pseudomonas aeruginosa* and the Enterobacter species.^7^

### 1.1 *Enterococcus faecium* (*E. faecium*)

These Gram-positive commonly chained or paired bacteria are frequented in the human and animal guts. Of the 20 odd species in the enterococcus family, *E. faecium* and *E. faecalis* are the common isolates.^8^^,^^9^ *Enterococcus* can cause ulcerative colitis, inflammatory bowel disease, and Crohns disease. It is notorious for high rates of cross-infection occurring through inanimate objects. Linezolid alone or in combination with gentamicin, doxycycline, or rifampicin is the standard of treatment.^10^.

### 1.2 *Staphylococcus aureus* (*S. aureus*)

These Gram-positive cocci are normal commensals of the skin and nostrils.^5^ They are associated with a high transmission rate occurring by direct contact or airborne spread. Dermal infections and pyogenic lesions affecting multiple organs are known to be caused by *S. aureus*.^11^^,^^12^ Community-acquired methicillin-resistant *S. aureus* (CA-MRSA) cases are a worrisome global affliction and are often lethal and usually treated by cephalosporins and oxacillin ^13^.

### 1.3 *Klebsiella pneumonia* (*K. Pneumonia*)

*K. pneumoniae* a non-fastidious, encapsulated Gram-negative bacillus are the most common bacterial pathogens encountered in the hospital caused by an infected host or they can be endogenous.^5^^,^^10^ It is usually associated with urinary tract infections and is best treated with third- and fourth-generation cephalosporins, quinolones, or carbapenems.^10^

### 1.4 *Acinetobacter baumannii* (*A. baumannii*)

*A. baumannii* is a Gram-negative, non-fermentative coccobacillus that causes infections in the respiratory and urinary systems, among other places. It is known to be sensitive in varying degrees to Meropenem and Colistin.^14^^,^^15^.

### 1.5 *Pseudomonas aeruginosa* (P.aeruginosa)

The facultative anaerobe, Gram-negative and rod-shaped *P.aeruginosa*, is found in the digestive tracts of humans. It is a common nosocomial, opportunistic bacterium that can cause serious infections with a high death rate in the immune-compromised. Owing to its adaptive antibiotic resistance, it is also one of the most difficult infections to treat. Aminoglycoside with beta-lactam penicillin is usually considered to be the first line treatment.^7^^,^^11^

### 1.6 Enterobacter species

*Enterobacter. spp* are Gram-negative, non-fastidious, rod shaped bacteria. One of the most common members of the *Enterobacteriaceae* family is *E. coli.* This pathogen can cause severe nosocomial illness in immunocompromised patients.^10^ including infections in the lower respiratory, ophthalmic, urinary and digestive tract, skin and soft-tissue and osteomyelitis.^16^

Possible treatments include carbapenems, fluoroquinolones, aminoglycosides and sulfamethoxazole/trimethoprim.^6^

The emergence of these resistant bacteria in orofacial infections leads to an increased morbidity and mortality leading to possible sequelae of life-threatening complications like airway obstruction, mediastinitis, necrotizing fascitis, cavernous sinus thrombosis, sepsis and thoracic empyema.^17^

The mainstay of treatment of odontogenic orofacial infections remains elimination of the nidus of infection, incision and drainage of pus from the facial spaces and effective antibiotic regimen. The empirical antibiotics used for the treatment of such infections area are amoxicillin-clavulanic acid, metronidazole and erythromycin. These pathogens however report resistance to amoxicillin in 9 to 54% of cases.^18^ What makes it more complex is that they are also resistant to a plethora of other higher antibiotics as well. Therefore, identification of the causative microorganism and aptly addressing the same medically remains a challenge when these multidrug resistant pathogens are involved.

This study was therefore aimed to investigate the presence of ESKAPE pathogens in association with orofacial infections, determine their antibiotic sensitivity and thereby establish viable treatment options for improved prognosis of the patients.

## 2 Materials and methods

Hundred and thirty two patients referred to the department of Oral and Maxillofacial Surgery, over a period of thirty five months (August 2019 to June 2022) with specific complaints of infectious symptoms diagnosed as orofacial space infection of odontogenic origin involving primary as well as secondary spaces. irrespective of their age and sex requiring hospital admission for treatment were included in this prospective clinical study. The study was approved by the Institutional ethics committee and written informed consent was obtained from all the participants for the collection of their data for this study. Data about the age, sex and medical comorbidities of the participants were collected using questionnaires.

After routine clinical and radiographic investigations, all patients underwent the following treatment protocol: The foci of infection/causative teeth identified and was extracted under local anesthesia followed by incision and drainage extra orally. Stab incision was made at most dependent area of the extraoral swelling and the first expelled pus was collected using sterile cotton swabs (Hiculture collecting device, HiMedia, Mumbai, India) and immediately transferred microbiology department for further investigation. After meticulous exploration of all the facial spaces involved via Hilton's method, corrugated drains were placed in situ for 48 h. All patients were started with empirical antibiotics of Amoxicillin 500 mg + potassium clavulanate 125 PO q 12 hourly.^3^^,^^19^ and Metronidazole PO 400 mg q 8 hourly. Severely ill patients, however, were started with Cefotaxime IV 1 g 12 hourly and Metronidazole IV 500 mg 8 hourly.^3^ After culture and sensitivity appropriate antibiotic therapy curated for the patient were administered.

Two sterile cotton swabs soaked in pus from the sample site was sent for culture and sensitivity testing.

One swab is utilized for gram staining on a glass slide while the other swab is inoculated by streak plate method in two agar media namely, Blood agar media (Himedia, Mumbai, India) and Mac Conkey agar media (Himedia, Mumbai, India) and both are incubated for around 6-12 h. It was noted that even the facultative anaerobes among ESKAPE pathogens (*E. faecium*, *K. pneumoniae*, *P. aeruginosa* and the Enterobacter spp.) can be cultured in an aerobic media owing to their ability to survive in the presence of oxygen and hence can be isolated from the site without them being destroyed in contact with oxygen.

Once the growth of microbial colony was noted, they were subjected for organism identification via the Matrix Assisted Laser Desorption Ionisation- Time of Flight Mass Spectrometry (Biomerieux, Marcy l’ Etoile, France).

Antibiotic sensitivity was then carried out using the VITEK 2 COMPACT (Biomerieux, Marcy l’ Etoile, France) system where the MIC (minimum inhibitory concentration) was determined by comparing the growth of the patient isolate to the growth of isolate with known MICs and the final report is generated. The number of isolates tested for each antibiotic varied because the VITEK 2 Compact system applies organism-specific antimicrobial panels according to CLSI guidelines. Consequently, not every antibiotic was tested against every bacterial isolate, resulting in different denominators across susceptibility analyses.

Antibiotic susceptibility testing was done for the following:

For the antibiotic susceptibility test: Amoxicillin-clavulanic acid, piperacillin-tazobactam were used as antibiotics containing β-lactam inhibitors; Aminoglycoside-based antibiotics amikacin and gentamicin; Cephalosporin-based cefuroxime, ceftriaxone, cefoperazone/sulbactam and cefepime; Carbapenem-based ertapenem, meropenem and imipenem; Lincosanide-clindamycin; Glycopeptide-based vancomycin; Macrolide-erythromycin; Quinolone-based ciprofloxacin and levofloxacin; Trimethoprim-sulfamethoxazole, Linezolid and Tetracycline were used.

Measurement of the inhibition zone was taken and interpreted as susceptible, intermediate and resistant based on CLSI guidelines 2020.^20^

The patients were administered the antibiotic drug regimen to which they tested sensitive. In cases where the patients did not respond to the first line of antibiotics, they were shifted to the other antibiotics tested positive. They were considered to be responding to the treatment when the symptoms of infection such as pus discharge, swelling, pyrexia were on the downward trend and when mouth opening and sustenance improved. The drains placed were removed when the drain volume was less than 25 ml over 24 h of continuous monitoring.

Patients were monitored for treatment outcomes throughout the period of hospitalization and discharged after complete recovery.

### 2.1 Statistical analysis

Data were analyzed using SPSS 20.0 (IBM Corp., Armonk, NY, USA). Frequencies and percentages were calculated for categorical variables. Comparisons between categorical variables were performed using the Chi-square test or Fisher's Exact test where appropriate. Continuous variables were assessed for normality. Hospitalization duration was compared between ESKAPE-positive and ESKAPE-negative groups using the Mann–Whitney *U* test. Statistical significance was set at p < 0.05.

## 3 Results

ESKAPE pathogens were isolated in 29 samples from the 132 samples procured. The prevalence rate of these pathogens in orofacial infections was found to be 21.97%. Enterobacter spp. (n = 10) was the most common bacteria followed by *K. pneumoniae* (n = 9), *S. aureus* (n = 6), *P. aeruginosa* (n = 2), and *A. baumannii* (n = 2). *E. faecium* was not detected. Cases of two or more causative ESKAPE occurring together were observed in 2 patients (*S. aureus* and Enterobacter spp., *K. pneumoniae* and Enterobacter spp. respectively)

Among the twenty nine *ESKAPE isolates* 18(62.07%) were from males and 11(37.93%) from females. However, there were no significant differences (p = 0.712) in sex predilection when compared to those in whom ESKAPE pathogens were negative where there were 63(55.75%) and 50 female (44.25%).

**Fig. 1:**
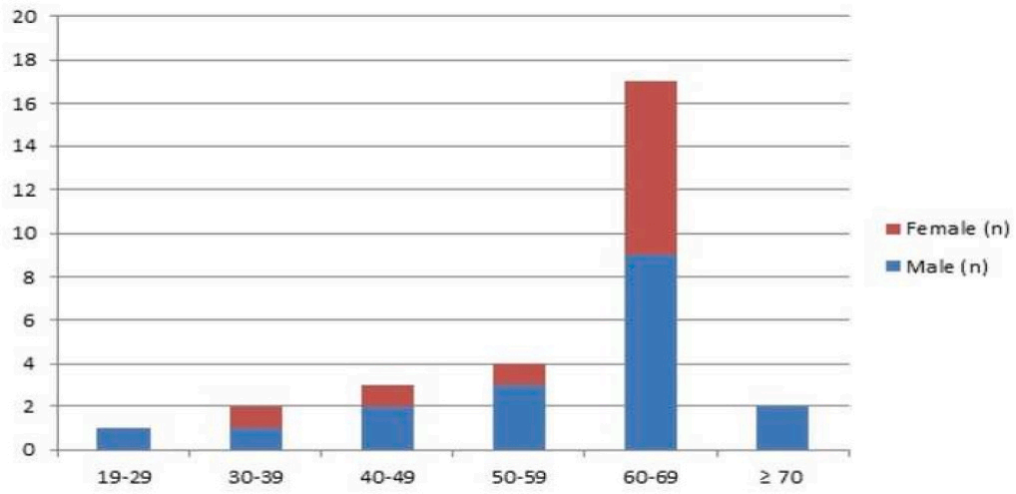
Age/sex distribution in ESKAPE positive population.

The age distribution of the entire study population, including both ESKAPE-positive and ESKAPE-negative groups, is presented in Table 1. The average age among the positive isolates population was 54.67 years. On comparison using Fisher's Exact test, patients aged 60–69 years had a significantly higher incidence of at least one ESKAPE pathogen compared to those below 60 years (p = 0.047). (Table 1, Fig. 1).

**Table 1:** Age distribution among the ESKAPE positive population (n = 29).

Age (years)	Male		Female		Total	
	n	%	n	%	n	%
19-29	1	5.55	-	-	1	3.45
30-39	1	5.55	1	9.09	2	6.90
40-49	2	11.11	1	9.09	3	10.34
50-59	3	16.68	1	9.09	4	13.79
60-69*	9	50.00	8	72.73	17	58.62
≥70	2	11.11	-		2	6.90
Total	18		11		29	

*p < 0.05 is statistically significant.

Treatment duration was categorized as ≤7 days, 8–14 days, 15–21 days, 22–28 days, and >29 days. Patients with ESKAPE-positive infections demonstrated significantly longer hospitalization compared with ESKAPE-negative patients (p = 0.021). More than half (55.17%) of ESKAPE-positive patients required hospitalization for 22–28 days compared with only 12.62% of ESKAPE-negative patients, while 13.79% of ESKAPE-positive patients required hospitalization for more than 29 days compared with 0.98% of ESKAPE-negative patients (Table 2). Patients with ESKAPE-positive infections had significantly longer hospitalization than ESKAPE-negative patients (p = 0.021). A greater proportion of ESKAPE-positive patients required hospitalization for 22–28 days (55.17%) and >29 days (13.79%), whereas most ESKAPE-negative patients (67.96%) were discharged within 8–14 days.

**Table 2:** Comparison of Duration of Treatment between ESKAPE negative and positive population.

Avg. Days of hospitalization	ESKAPE negative (n = 103)		ESKAPE positive (n = 29)	
	n	%	n	%
≤7 days	2	1.94	-	-
8- 14 days	70	67.96	3	10.34
15-21 days	17	16.50	6	20.69
22-28 days	13	12.62	16	55.17*
>29 days	1	0.98	4	13.79*

*p < 0.05 is statistically significant.

Although based on the antibiotic susceptibility test, the ESKAPE group showed resistance to the empirical antibiotic in 17 cases antibiotics were not changed in 5 patients whose symptoms were relieved by the initial treatment. The other patients were prescribed a change in antibiotics based on the test results. Table 3 summarizes the results of the antibiotic susceptibility tests for ESKAPE strains.

**Table 3:** Antibiotic susceptibility of isolated ESKAPE microorganisms.

Antibiotic	*Staphylococcus aureus (n=6)*			*Klebsiella pneumoniae (n=9)*			*Acinetobacter baumannii (n=2)*			*Pseudomonas aeruginosa (n=2)*			*Enterobacter* spp. (n = 10)			Total Isolates tested (N)	Overall		
	N (%)			N (%)			N (%)			N (%)			N (%)				N (%)		
	S	I	R	S	I	R	S	I	R	S	I	R	S	I	R		S	I	R
Amoxicillin + Clavulanic Acid	4(66.66)	1(16.67)	1(16.67)	2(22.22)		7(77.78)			2(100)	2(100)			1(10)	1(10)	2(80)	29	9(31.03)	2(6.90)	18(62.07)
Piperacillin-Tazobactam	2(33.34)	4(66.66)		6(66.67)		3(33.33)			2(100)	2(100)			7(70)		3(30)	29	17(58.62)	4(13.79)	8(27.58)
Amikacin				7(77.78)	2(2.22)				2(100)	2(100)			10(100)			23	19(82.60)	4(8.70)	4(8.70)
Gentamicin				5(55.56)	2(22.22)	2(22.22)			2(100)	2(100)			10(100)			23	17(73.91)	2(8.70)	4(17.39)
Cefuroxime	5(83.33)	1(16.67)													10(100)	16	5(31.25)	1(6.25)	10(62.5)
Ceftriaxone,	6(100)			8(88.89)	1(11.11)				2(100)	1(50)	1(50)		7(70)		3(30)	29	22(75.86)	2(6.90)	5(17.24)
Cefoperazone/Sulbactam									2(100)	1(50)	1(50)		8(80)	2(20)		14	9(64.29)	3(21.42)	2(14.29)
Cefepime									2(100)	2(100)			9(90)	1(10)		14	11(78.57)	1(7.14)	2(14.29)
Ertapenem										2(100)			10(100)			12	12(100)		
Meropenem				2(22.22)		7(77.78)			2(100)	2(100)			9(90)	1(10)		23	13(56.52)	1(4.35)	9(39.13)
Imipenem				9(100)					2(100)	2(100)			9(90)	1(10)		23	20(86.96)	1(4.35)	2(8.69)
Clindamycin	6(100)															6	6(100)		
Vancomycin	5(83.33)		1(16.67)													6	5(83.33	1(16.67)	
Erythromycin	6(100)								2(100)							8	6(75)		2(25)
Ciprofloxacin				1(11.11)		8(88.89)			2(100)	2(100)			10(100)			23	13(56.52)		10(43.48)
Levofloxacin	5(83.33)		1(1.67)						2(100)	2(100)			10(100)			20	17(85)		3(15)
Trimethoprim-Sulfamethoxazole	5(83.33)		1(1.67)	1(1.11)		8(88.89)			2(100)			2(100)				19	6(31.58)		13(68.42)
Linezolid	6(100)															6	6(100)		
Tetracycline		1(16.67)	5(83.33)	7(77.78)	2(22.22)				2(100)	1(50)	1(50)					19	8(42.10)	4(21.05)	7(36.84)

X: Total isolates tested against the specific antibiotic *p < 0.05 is statistically significant.

Using Fisher's Exact test, the association between the isolates and antibiotic susceptibility were analyzsed. *Staphylococcus aureus* isolates showed the highest overall susceptibility and were significantly sensitive to cephalosporins (p = 0.047) and β-lactam/β-lactamase inhibitors (p = 0.048, Fisher's Exact test).

*Klebsiella pneumoniae* isolates were significantly sensitive to aminoglycosides (p = 0.036) and carbapenems (p = 0.041). *Acinetobacter baumannii* isolates were resistant to all tested antibiotics. *Pseudomonas aeruginosa* isolates were sensitive to all antibiotics tested except trimethoprim-sulfamethoxazole.

Enterobacter spp. isolates were significantly sensitive to aminoglycosides (p = 0.025), carbapenems (p = 0.028), and quinolones (p = 0.037), but showed the highest resistance to amoxicillin-clavulanic acid.

The highest susceptibility overall was observed for linezolid, ertapenem, and clindamycin (100%), followed by imipenem (86.96%), levofloxacin (85%), and amikacin (82.60%), whereas trimethoprim-sulfamethoxazole (68.42%), cefuroxime (62.5%), and amoxicillin-clavulanic acid (62.07%) had the highest resistance rates.

Systemic comorbidities were observed in 51 (38.64%) of the 132 patients. They were significantly more frequent among patients with ESKAPE-positive infections (19/29; 65.52%) than among ESKAPE-negative patients (32/103; 31.07%) (p = 0.039). Among the ESKAPE-positive patients with comorbidities, diabetes mellitus was the most common condition (42.10%), followed by hypertension (21.06%), cancer (15.78%), immunodeficiency disorders (10.35%), and other cardiac, neurological, renal, or hepatic disorders (10.35%) (Table 4).

**Table 4:** Distribution of systemic comorbidities among the study population.

Variable	ESKAPE Positive (n = 29)	ESKAPE Negative (n = 103)	Total (n = 132)	p-value
Patients with ≥1 systemic comorbidity	19 (65.52%)	32 (31.07%)	51 (38.64%)	0.039*
Patients without systemic comorbidity	10 (34.48%)	71 (68.93%)	81 (61.36%)	—
**Comorbidity profile among ESKAPE-positive patients**				
Diabetes mellitus	8 (42.10%)	NR	8	—
Hypertension	4 (21.06%)	NR	4	—
Cancer	3 (15.78%)	NR	3	—
Immunodeficiency disorders	2 (10.35%)	NR	2	—
Cardiac/Neurological/Renal/Hepatic disorders	2 (10.35%)	NR	2	—

Values are presented as n (%). NR = Not reported, as the distribution of individual comorbidities among the ESKAPE-negative group was not available in the study dataset. *p < 0.05 was considered statistically significant.*

A detailed table of antibiotic dosages and administration schedules has been included to enhance the clinical applicability of the study and guide evidence-based therapeutic decisions. Dosages represent standard adult regimens. Final antibiotic selection, dose adjustments, and duration were individualized based on culture sensitivity results, patient clinical response, renal/hepatic status, and institutional protocols (Table 5).

**Table 5:** Detailed dosages and administration schedules of the individualized treatments.

Clinical scenario	Antibiotic regimen	Adult dosage*	Route	Frequency	Remarks
Empirical therapy (mild to moderate odontogenic orofacial infection)	Amoxicillin + Clavulanic acid	500 mg/125 mg	Oral	Every 12 h	First-line empirical therapy initiated at admission in clinically stable patients.
	Metronidazole	400 mg	Oral	Every 8 h	Administered in combination with amoxicillin-clavulanic acid to provide anaerobic coverage.
Empirical therapy (severe infection requiring hospitalization)	Cefotaxime	1 g	Intravenous	Every 12 h	Administered to patients presenting with severe infection or systemic involvement.
	Metronidazole	500 mg	Intravenous	Every 8 h	Combined with cefotaxime for anaerobic coverage.
Culture-directed therapy	Antibiotic selected according to antimicrobial susceptibility testing	Individualized	Oral/Intravenous	According to selected agent	Therapy was modified based on organism identification, antimicrobial susceptibility profile, clinical response, and patient-specific factors.
Commonly susceptible antibiotics identified in the present study	Imipenem	500 mg	Intravenous	Every 6 h	Demonstrated high overall susceptibility (86.96%) among ESKAPE isolates.
	Ertapenem	1 g	Intravenous	Once daily	Demonstrated 100% susceptibility among tested isolates.
	Linezolid	600 mg	Oral/Intravenous	Every 12 h	Demonstrated 100% susceptibility in susceptible Gram-positive isolates.
	Clindamycin	600 mg	Intravenous	Every 8 h	Demonstrated 100% susceptibility among tested susceptible isolates.
	Amikacin	15 mg/kg/day	Intravenous	Once daily	High susceptibility, particularly against Gram-negative isolates.
	Levofloxacin	500–750 mg	Oral/Intravenous	Once daily	Demonstrated high susceptibility against susceptible isolates.

## 4 Discussion

Antibiotic resistance is a clinical conundrum that poses great threat to treatment outcomes in terms of both prognosis and associated mortality and morbidity. The multidrug resistant ESKAPE pathogens in particular are infamous for a multitude of antibiotic resistance patterns ranging from modifying drug pharmacodynamics, drug efflux to complete drug inactivation.^5^^,^^10^^,^^17^ Therefore infections involving these pathogens is not only severe but do not respond to treatment and may ensue life threatening complications. It is therefore necessary to constantly evolve our antibiotic arsenal accordingly. Over the years, the prevalence ESKAPE bacteria in the maxillofacial region infections have tremendously increased mandating that we must not neglect these pathogens in the routines.^17^ This study was conducted to evaluate the risk of infection by ESKAPE pathogens in odontogenic orofacial infections and their respective antibiotic susceptibility.

Of the total 132 patients of odontogenic orofacial infection reporting to our unit in nearly 3 year period, ESKAPE strains were isolated from 29 patients (21.97%). The results are in congruence with other previous studies, reiterating that these pathogens are becoming more frequent to the oral and maxillofacial region and pose serious threats to the prognostic outcomes, hence are not to be neglected in our diagnostic radar ^12^^,^^21^, ^22^, ^23^, ^24^.

The study showed that these pathogens frequented in the elderly (>60 years) in comparison to those that were younger. 65.52% of the patients with ESKAPE isolates had other associated systemic comorbidities. Both these results are in line with other studies and reiterate that these pathogens are leading causes of opportunistic and nosocomial infections and are a definite cause for concern in orofacial infections of susceptible individuals. 25, 26, 27.

Our study finds that the increase in the average days of hospitalization and antibiotic resistance associated with ESKAPE infections are in tandem to each other 28, 29, 30 which agrees with the findings of previous studies. Antibiotics resistance can also be attributed also to prolonged periods of drug administration of multiple drug regimens.^29^^,^^30^ Early diagnosis and swift identification of the presence of these multidrug resistant pathogens in orofacial infections holds the key to appropriate antibiotic selection thereby improving the prognosis and reducing the recuperation period of the patient. 31, 32, 33.

The study results indicate that Enterobacter sp. was the most common bacteria (34.48%) followed by *K. pneumoniae* (31.03%). However, other studies have reported that *K. pneumoniae* was the most prevalent pathogen followed by *S. aureus*.^6^^,^^10^^,^^11^ This variation in distribution pattern of the pathogen could be attributed to geographical distribution, microbial competition, host factors and antibiotic resistance.

In the present study, there were variations in the antibiotic susceptibility patterns of the bacterial isolates. Linezolid, ertapenem and clindamycin were the most effective antibiotics against most isolates, followed imipenem, levofloxacin and amikacin. These findings are similar to those of Ramsamy Y et al.^18^ Also noticed was that the majority of the isolates were highly resistant to Trimethoprim-sulfamethoxazole, cefuroxime and amoxicillin-clavulanic acid as seen in the findings of De Angelis G et al. and other similar studies.^16^^,^^22^^,^^23^

According to previous studies, Enterobacter spp. showed relatively high resistance to penicillin, clindamycin, and fluoroquinolone-based antibiotics and *K. pneumoniae* has a 56% to 75% resistance rate to cephalosporin and a high sensitivity to piperacillin and amikacin.^10^^,^^12^ In our study, Enterobacter spp. showed very high resistance to amoxicillin-clavulanic acid but was significantly sensitive to aminoglycosides, carbapenems and quinolones and *K. pneumoniae* were significantly sensitive to aminoglycosides and carbapenem group of antibiotics.

*P. aeruginosa* was susceptible to antibiotics such as amikacin, tobramycin, piperacillin-tazobactam, meropenem and colistin in previous study,^18^ but this study showed 100% sensitivity to all antibiotics. *A. baumannii*, however, was 100% resistant to all antibiotics tested against. In other studies, by Jones et al. and Jellison et al.,^34^ *A. baumannii* showed sensitivity to tetracycline and sulbactam respectively. However, it must be noted that *A. baumannii* and *P. aeruginosa* were identified in only two cases each in our study and hence reliability is poor.

This study offers several key strengths. By conducting a region-specific analysis of ESKAPE pathogens in odontogenic infections, it addresses an important knowledge gap in the Indian context. The use of a comprehensive antibiotic panel provides clinically actionable susceptibility patterns, while the application of Fisher's Exact test ensures statistical validity even with small sample sizes. The study also identifies correlations between age, comorbidities, and pathogen prevalence, which can guide risk stratification and early intervention strategies. Furthermore, it presents detailed, pathogen-specific resistance and susceptibility profiles that can directly inform evidence-based antibiotic stewardship in oral and maxillofacial infections. Collectively, these findings reinforce the importance of early detection and targeted therapy, highlighting the practical clinical relevance of the results.

Although many studies worldwide have identified antibiotics that are effective against the ESKAPE pathogens, applying these results domestically is ambiguous owing to environmental, genetic, and social factors. Also the outcomes on respiratory or urinary infections may not be applicable in oral and maxillofacial infections. Hence this study may provide insight into the same in an Indian context. The strengths of the present study include its prospective design, the standardized microbiological identification using MALDI-TOF MS and VITEK 2 COMPACT and systematic antimicrobial susceptibility testing, which provide reliable microbiological and resistance data in patients with odontogenic infections can be identified as another strength of the study.

There are however a few limitations in our clinical study. Our study included a small number of subjects in a single institution and the various antibiotics were not consistently applied to the detected strains during the antibiotic susceptibility test, making it difficult to analyze and compare various antibiotic resistance rates for each bacterium. Since it was conducted at a single tertiary care center with a relatively small sample size, this may have limited the generalizability of the findings. Multicenter studies involving larger populations are required to validate these observations. Another limitation of the present study is that antimicrobial susceptibility testing was performed using organism-specific panels generated by the automated VITEK 2 Compact system. Consequently, not all antibiotics were tested against every isolate, resulting in varying denominators for susceptibility analysis and limiting direct comparison of resistance rates across all bacterial species.Both *Acinetobacter baumannii* isolates identified in the present study demonstrated resistance to all antibiotics tested. However, as only two isolates were identified, this finding should be interpreted with caution and cannot be considered representative of the broader resistance profile of *A. baumannii*. Therefore to eliminate these limitations and increase the reliability of the results, further research must include larger population pool and ensure consistent antibiotic testing against each pathogen.

## 5 Conclusion

In conclusion, the findings of our study underscore the critical importance for clinicians to remain vigilant about the potential involvement of ESKAPE pathogens in orofacial infections. Given the multidrug-resistant nature of these organisms and their increasing prevalence, it is imperative to perform culture and sensitivity testing in all patients, regardless of disease severity or stage. This approach enables the selection of targeted, evidence-based antibiotic therapies tailored to individual patient needs, which not only improves treatment efficacy but also minimizes the risk of complications, prolonged hospitalization, and other adverse outcomes. Additionally, consistent follow-up and reassessment of antibiotic regimens are essential to ensure optimal patient recovery and to prevent the emergence of further resistance. Ultimately, incorporating these practices into routine clinical management can enhance prognostic outcomes, reduce morbidity, and support more judicious use of antibiotics in the management of odontogenic and orofacial infections.

## Ethical clearance obtained

Number: Ethics/ABSMIDS/330/2023.

## Consent

Obtained from patients.

## Funding

This research did not receive any specific grant from funding agencies in the public, commercial, or not-for-profit sectors.

## Declaration of competing interest

The authors declare that they have no known competing financial interests or personal relationships that could have appeared to influence the work reported in this paper.
